# Broadening the infection prevention and control network globally; 2017 Geneva IPC-think tank (part 3)

**DOI:** 10.1186/s13756-019-0528-0

**Published:** 2019-05-10

**Authors:** Walter Zingg, Julie Storr, Benjamin J. Park, John A. Jernigan, Stephan Harbarth, M. Lindsay Grayson, Evelina Tacconelli, Benedetta Allegranzi, Denise Cardo, Didier Pittet, the 2017 Geneva-IPC Think Tank, Mohamed Abbas, Raheelah Ahmad, Benedetta Allegranzi, Antoine Andremont, Mike Bell, Michael Borg, Denise Cardo, Yehuda Carmeli, Enrique Castro-Sanchez, John Conly, Philippe Eggimann, Petra Gastmeier, M. Lindsay Grayson, Stephan Harbarth, Marcela Hernandez, Loreen Herwaldt, Alison Holmes, John A. Jernigan, Claire Kilpatrick, Amy Kolwaite, Karl-Heinz Krause, Elaine Larson, Sarah Masson-Roy, Shaheen Mehtar, Marc Mendelson, Ling Moi Lin, Andreea Moldovan, Dominique Monnet, Babacar Ndoye, Peter Nthumba, Folasade Ogunsola, Ben Park, Eli Perencevich, Didier Pittet, Matthew Samore, Wing Hong Seto, Arjun Srinivasan, Julie Storr, Evelina Tacconelli, Carolyn Tarrant, Sara Tomczyk, Maha Talaat, Maria Virginia Villegas, Andreas Voss, Tim Walsh, Andreas Widmer, Walter Zingg

**Affiliations:** 1 Infection control programme and WHO collaborating center, University of Geneva Hospitals and Faculty of Medicine, 4 Rue Gabrielle Perret-Gentil, 1211 Geneva 14, Switzerland; 2 Infection Prevention and Control Global Unit, World Health Organization, Geneva, Switzerland; 3 US Centers for Disease Control and Prevention, Atlanta, GA USA; 4 Infectious Diseases Department, Austin Health, University of Melbourne, Heidelberg, Victoria Australia; 5 Infectious diseases unit, University Hospital Tübingen, Tübingen, Germany; 6 Department of diagnostics and public health, University of Verona, Verona, Italy

**Keywords:** Networks, Collaboration, Infection prevention and control, International, National, Institutional, Change, CDC, ECDC, WHO

## Abstract

**Background:**

Healthcare-associated infection (HAI) is a major challenge for patient safety worldwide, and is further complicated by antimicrobial resistance (AMR) due to excessive antimicrobial use in both humans and animals. Existing infection prevention and control (IPC) networks must be strengthened and adapted to better address the global challenges presented by emerging AMR.

**Methods:**

In June 2017, 42 international experts convened in Geneva, Switzerland, to discuss two key areas for strengthening the global IPC network: 1) broadening collaboration in IPC; and 2) how to bring the fields IPC and AMR control together.

**Results:**

The US Centers for Disease Prevention and Control, the European Centre for Disease Prevention and Control, and the World Health Organization (WHO) convened together with international experts to discuss collaboration and networks, demonstrating the participating organizations’ commitment to close collaboration in IPC. The challenge of emerging AMR can only be addressed by strengthening this collaboration across international organisations and between public health and academia. The WHO *SAVE LIVES: Clean Your Hands* initiative is an example of a successful collaboration between multiple global stakeholders including academia and international public health organisations; it can be used as a model. IPC-strategies are included within the four pillars to combat AMR: surveillance, IPC, antimicrobial and diagnostic stewardship, research and development. The prevention of transmission of multidrug-resistant microorganisms is a patient safety issue, and must be strengthened in the fight against AMR.

**Conclusions:**

The working group determined that international organisations should take the lead in creating new networks, which will in turn attract academia and other stakeholders to join. At the same time, they should invest in bringing existing IPC and AMR networks under one umbrella. Transmission of multidrug-resistant microorganisms in hospitals and in the community threatens the success of antimicrobial stewardship programmes, and thus, research and development in IPC should be addressed as an enhanced global priority.

## Background

Under the auspices of the US Centers for Disease Control and Prevention (CDC), the World Health Organization (WHO), and the University of Geneva Hospitals and Faculty of Medicine (HUG), an international panel of 42 experts with backgrounds in infection prevention and control (IPC), microbiology, infectious diseases, public health, psychology, medical technology, and social sciences, convened for 2 days at the Geneva Think Tank on IPC and antimicrobial resistance (AMR) in June 2017. The aim was to develop a global vision on IPC and AMR, and to agree on a road map for research and public health activities. Three dimensions of IPC and AMR were discussed: 1) implementation of IPC and antimicrobial stewardship; 2) technology in IPC and AMR; and 3) broadening the global IPC network. This is the final paper in a series of three summarising the discussions during the meeting, and addressing the current and future role of networks in IPC.

## Methods

Two key areas for strengthening the IPC networks globally were addressed: 1) broadening collaboration among IPC organisations around the world; and 2) improving and aligning IPC and AMR control efforts. Two impulse talks for each of the areas set the stage for a moderated plenary discussion. Writers took notes. This paper is the summary of presentations and notes taken from the plenary discussion.

## Results

CDC, the European Centre for Disease Prevention and Control (ECDC), and the WHO convened together and with international experts to exchange their visions about collaboration and networking, and demonstrated their commitment for close collaboration in IPC.

### Broadening the collaboration in infection prevention and control and antimicrobial resistance

A number of international IPC and AMR networks are already in place (Table 1). The WHO Global IPC Network brings together major international and national IPC organisations and in 2017, issued a call to action related to the global and national IPC priorities for 2018–2022 [1]. Pre-existing surveillance modules on AMR, antimicrobial consumption, and HAI surveillance in Europe have been integrated to become the ECDC ARHAI-Net (Antimicrobial resistance and healthcare-associated infection network). This network collaborates with a number of other networks. The Trans-Atlantic Task Force for Antimicrobial Resistance (TATFAR) is an example of a transatlantic collaboration between a number of European and US members (Table 1). The initiative agreed on 17 recommendations in 3 key areas: appropriate therapeutic use of antimicrobial drugs in medical and veterinary communities; prevention of drug-resistant infections; strategies for improving the pipeline of new antimicrobial drugs and diagnostic devices, and maintaining existing drugs on the market. There was consensus in the group that the natural stakeholders for international collaboration are CDC, ECDC, and WHO, although other regional or global organisations should also be engaged. These public health organisations work on the political level, cascading ideas and policies down to ministries of health.

**Table 1:** Collaborative networks in infection prevention and control and antimicrobial resistance on national and international levels

Acronym	Network	Lead	Description/Mission	Links
ARHAI-Net	Antimicrobial Resistance and Healthcare-Associated Infections Network	ECDC	Focus on surveillance, scientific advice, training and communication to address the threat of antimicrobial resistance and healthcare-associated infections. ARHAI integrated the former European programmes ESAC on antimicrobial consumption, EARSS on antimicrobial resistance, HELICS on healthcare-associated infections (surgical site infections; HAI in intensive care), and IPSE on a wider patient safety context.	https://ecdc.europa.eu/en/about-us/who-we-are/disease-programmes/antimicrobial-resistance-and-healthcare-associated
EIP	Emerging Infections Program	US-CDC	Prevention and control of infectious diseases by providing scientific information to monitor emergency problems, evaluate public health interventions, and inform policy.	https://www.cdc.gov/ncezid/dpei/eip/index.html
IMI	Innovative Medicines Initiative	EC & EFPIA	Working group on accelerating development and access to innovative medicines, particularly in areas where there is an unmet need. IMI is the world’s biggest public-private partnership in life sciences.	https://www.imi.europa.eu/about-imi/mission-objectives
GIPCN	Global Infection Prevention and Control Network	WHO	GIPCN aims to enhance local, national and international coordination and collaboration in the field of IPC and to support WHO’s and countries’ efforts on IPC, from preparedness, to IPC systems and programmes’ strengthening, outbreak prevention and control, as well as capacity building for surveillance.	http://www.who.int/infection-prevention/about/GIPC_Network/en/
GLASS	Global Antimicrobial Resistance Surveillance System	WHO	GLASS promotes and supports a standardized approach to the collection, analysis and sharing of AMR data at a global level by encouraging and facilitating the establishment of national AMR surveillance systems that are capable of monitoring AMR trends and producing reliable and comparable data.	https://www.who.int/glass/partnerships/en/
JPIAMR	Joint Programming Initiative on Antimicrobial Resistance	EU	Basic and exploratory research on new antibiotics, stewardship of existing antibiotics, and control of the spread of antibiotic resistance between humans, animals, and the environment in a One Health perspective (26 countries globally).	https://www.jpiamr.eu/
POPS	Private Organizations for Patient Safety	WHO	Public-private partnership to harness industry strengths to align and improve implementation of WHO recommendations in different parts of the world.	http://www.who.int/infection-prevention/about/pops/en/
TATFAR	Trans-Atlantic Force for Antimicrobial Resistance	US-CDC	Transatlantic collaboration between US-CDC, ECDC, EC, EFSA, FDA, AMA, OGHA, and USDHS. Improving appropriate therapeutic use of antimicrobial drugs in medical and veterinary communities; preventing healthcare- and community-associated drug-resistant infections, and developing strategies for improving the pipeline of new antimicrobial drugs.	https://www.cdc.gov/drugresistance/tatfar/index.html
	Antibiotic Resistance Solutions Initiative	US-CDC	The initiative invests in national infrastructure to detect, respond, contain, and prevent resistant infections across healthcare settings, food, and communities. CDC funding supports all 50 state health departments, six local health departments, and Puerto Rico.	https://www.cdc.gov/drugresistance/solutions-initiative/index.html
	Prevention EpiCenters	US-CDC	Collaborative research programme between the CDC’s division of healthcare quality promotion and academic investigators for conducting innovative infection prevention and control research.	https://www.cdc.gov/hai/epicenters/index.html
	WHO *SAVE LIVES: Clean Your Hands*	WHO	WHO’s global annual campaign making a call to action for health workers every 5 May concerning preventing HAI through hand hygiene and IPC improvements .	http://www.who.int/infection-prevention/campaigns/clean-hands/en/

*AMA* American Medical Association, *EARSS* European Antimicrobial Resistance Surveillance System, *EC* European Commission, *EFPIA* European Federation of Pharmaceutical Industries and Associations, *EFSA* European Food Safety Authority, *ESAC* European Surveillance on Antimicrobial Consumption, *EU* European Union, *FDA* US Food and Drug Association, *HAI* Healthcare-associated infection, *HELICS* Hospitals in Europe Link for Infection Control through Surveillance, *IPSE* Improving Patient Safety in Europe, *NIH* US National Institutes of Health, *OGHA* Office of Global Health Affairs, *US-CDC* US Centers for Disease Control and Prevention, *USDHS* US Department of Homeland Security, *WHO* World Health Organization

Experts in IPC and professional societies may not have a public health perspective, but the role they play in driving innovation and generating ideas and solutions cannot be dismissed given the impact of such efforts on future public health decision making. Ideally, expert-to-expert collaboration would include international public health bodies. Any resulting innovations would be more likely to incorporate global or higher-level policy perspectives, with the potential impact extending beyond research to become standard in patient care. The WHO *SAVE LIVES: Clean Your Hands* campaign exemplifies the success of such collaboration, where the campaign principle is that public health and IPC experts work hand-in-hand to drive improvement [2]. In addition, the WHO hand hygiene campaign benefits from the collaboration of a public-private partnership, developed under the umbrella of the WHO Private Organizations for Patient Safety (POPS)-Hand Hygiene. This collaboration, among others, enables campaign messaging to be spread at the bedside in many countries, using multiple languages, and adapting to different cultural and resource needs [2]. Through its collaborating centres, WHO has further strengthened the network between public health and academic institutions. The integration of Improving Patient Safety in Europe (IPSE), European Surveillance of Antimicrobial Consumption (ESAC), and European Antimicrobial Resistance Surveillance System (EARSS) under the umbrella of ECDC’s ARHAI-Net is another example of successfully transforming academic work into a large scale public health initiative. Examples from CDC’s numerous collaborations with academia include the Prevention EpiCenters and the Emerging Infections Program (EIP). There is little collaboration of the many private initiatives in IPC and AMR. As partners, international organisations can streamline and leverage the effect of such activities.

A challenge is the fact that research, and consequently the guidance based on scientific findings, is typically generated in high income countries. More research in low-and-middle-income countries (LMICs) is needed, but until this is available, at a minimum, the applicability of recommendations to LMIC contexts should be assessed. The WHO core components for IPC are a good example of guidelines based on data primarily from high income settings that have been integrated with experience and additional evidence from the field gathered in LMIC settings according to a solid methodology. In collaboration with CDC, WHO has developed implementation tools with special focus on low resource settings, to support the adaptation and adoption of the guidelines into the local context [3, 4]. These are examples on how international organisations can fill their role as trusted promoters in improving the implementation of IPC and AMR control in LMIC.

### How to bring IPC and antimicrobial resistance control together

Emerging AMR is a global problem, which can only be controlled if stakeholders are working together at an international level. AMR control relies on four pillars: 1) surveillance (of infections, resistance and antimicrobial use); 2) IPC (prevention of AMR transmission); 3) antimicrobial stewardship; and 4) research and development. It is well accepted that all four pillars are needed to both define the problem (research, surveillance), and to solve it (IPC, antimicrobial stewardship).

Preventing the spread of multidrug-resistant organisms depends predominantly on good IPC practice in healthcare facilities, particularly hand hygiene, institutional environmental cleaning, and isolation precaution measures. Prevention of spread in the community setting is also predicated on the existence of adequate water, sanitation and hygiene measures. Additional measures include screening of patients at risk for carriage of multi-resistant organisms according to defined strategies that are adapted to individual types of AMR-pathogen combinations. There is sufficient evidence that such strategies work, the recommended actions are straight-forward, and they can be applied immediately. If cross-transmission is not contained, other AMR strategies such as antimicrobial stewardship or the development of new antimicrobials will be very limited in both effect and sustained impact.

IPC as a core prevention measure has neither been sufficiently recognized nor prioritized at the global level to adequately address the scope of the AMR problem. The field of IPC is not adequately interconnected with the AMR community. This dichotomy is rooted in history. Research in AMR is infectious diseases and microbiology-driven, often focusing on resistance mechanisms and genetic relatedness. Infection prevention and control has a more practical attitude focusing on the prevention of cross-transmission by promoting best practice procedures. Scope and professional backgrounds of stakeholders in IPC and AMR are often different. However, the need for collaboration between the two has become more urgent. International organisations such as CDC, ECDC, and WHO could take a lead on bridging the gap between IPC and AMR experts. ECDCs ARHAI network and CDC’s Antibiotic Resistance Solutions Initiative are examples where activities on AMR and IPC are under the same umbrella.

## Discussion

International organisations should take the lead in both strengthening existing and creating new networks, which will attract academia and other stakeholders to join. At the same time, they should invest in bringing existing IPC and AMR networks under one umbrella. Transmission of multidrug-resistant microorganisms in hospitals and in the community threatens successful combating of AMR, and thus, measures to interrupt transmission i.e. effective IPC strategies and programmes supported by effective international and national networks should be given more priority globally.

## Conclusion

The working group determined that international organisations should take the lead in creating new networks, and invest in bringing existing IPC and AMR networks under one umbrella. Research and development in IPC should be addressed as an enhanced global priority.

## Abbreviations

AMA: American Medical Association
AMR: Antimicrobial resistance
ARHAI: Antimicrobial Resistance and Healthcare-Associated Infection (−network)
CDC: US Centers for Disease Control and Prevention
EARSS: European Antimicrobial Resistance Surveillance System
EC: European Commission
ECDC: European Centre for Disease Prevention and Control
EFPIA: European Federation of Pharmaceutical Industries and Associations
EFSA: European Food Safety Authority
EIP: (CDC) Emerging Infections Program
ESAC: European Surveillance on Antimicrobial Consumption
EU: European Union
FDA: US Food and Drug Association
HAI: Healthcare-associated infection
HELICS: Hospitals in Europe Link for Infection Control through Surveillance
HUG: University of Geneva Hospitals
IMI: Innovative Medicines Initiative
IPC: Infection prevention and control
IPSE: Improving Patient Safety in Europe
JPIAMR: Joint Programming Initiative on Antimicrobial Resistance
LMIC: Low-and-middle-income country
NIH: US National Institutes of Health
OGHA: Office of Global Health Affairs
POPS: Private Organizations for Patient Safety
TATFAR: Trans-Atlantic Task Force on Antimicrobial Resistance
USDHS: US Department of Homeland Security
WHO: World Health Organization
